# Modulation of microbial communities and mucosal gene expression in chicken intestines after galactooligosaccharides delivery *In Ovo*

**DOI:** 10.1371/journal.pone.0212318

**Published:** 2019-02-27

**Authors:** Anna Slawinska, Aleksandra Dunislawska, Arkadiusz Plowiec, Malgorzata Radomska, Jagoda Lachmanska, Maria Siwek, Siria Tavaniello, Giuseppe Maiorano

**Affiliations:** 1 Department of Animal Biotechnology and Genetics, UTP University of Science and Technology, Bydgoszcz, Poland; 2 Department of Agricultural, Environmental and Food Sciences, University of Molise, Campobasso, Italy; University of New England, AUSTRALIA

## Abstract

Intestinal mucosa is the interface between the microbial content of the gut and the host’s milieu. The goal of this study was to modulate chicken intestinal microflora by *in ovo* stimulation with galactooligosaccharides (GOS) prebiotic and to demonstrate the molecular responses of the host. The animal trial was performed on meat-type chickens (Ross 308). GOS was delivered by *in ovo* injection performed into the air cell on day 12 of egg incubation. Analysis of microbial communities and mucosal gene expression was performed at slaughter (day 42 post-hatching). Chyme (for DNA isolation) and intestinal mucosa (for RNA isolation) from four distinct intestinal segments (duodenum, jejunum, ileum, and caecum) was sampled. The relative abundance of *Bifidobacterium* spp. and *Lactobacillus* spp. in DNA isolated from chyme samples was determined using qPCR. On the host side, the mRNA expression of 13 genes grouped into two panels was analysed with RT-qPCR. Panel (1) included genes related to intestinal innate immune responses (*IL-1β*, *IL-10* and *IL-12p40*, *AvBD1* and *CATHL2*). Panel (2) contained genes involved in intestinal barrier function (*MUC6*, *CLDN1* and *TJAP1*) and nutrients sensing (*FFAR2* and *FFAR4*, *GLUT1*, *GLUT2* and *GLUT5*). GOS increased the relative abundance of *Bifidobacterium* in caecum (from 1.3% to 3.9%). Distinct effects of GOS on gene expression were manifested in jejunum and caecum. Cytokine genes (*IL-1β*, *IL-10* and *IL-12p40*) were up-regulated in the jejunum and caecum of the GOS-treated group. Host defence peptides (*AvBD1* and *CATHL2*) were up-regulated in the caecum of the GOS-treated group. Free fatty acid receptors (*FFAR2* and *FFAR4*) were up-regulated in all three compartments of the intestine (except the duodenum). Glucose transporters were down-regulated in duodenum (*GLUT2* and *GLUT5*) but up-regulated in the hindgut (*GLUT1* and *GLUT2*). In conclusion, GOS delivered *in ovo* had a bifidogenic effect in adult chickens. It also modulated gene expression related to intestinal immune responses, gut barrier function, and nutrient sensing.

## Introduction

Mucous tissue lines the gastrointestinal tract (GIT). It is composed of a vast surface area that forms an interface between luminal microflora and internal body structures. Mucosa is in constant cross-talk with the microbiome and reacts directly to signals from the gut environment [1]. The effects of such interaction on the host are immense: from intestinal health, improved digestion, metabolism regulation, and maturation of the immune system, to regulation of behaviour and body weight [2]. Composition of the microbiome can be altered by different environmental factors [3]. Some of them, including a low-fibre diet or use of antimicrobials or enteric infection agents, decrease the biodiversity and abundance of the beneficial microflora [3]. Others, such as prebiotics, probiotics, synbiotics, or postbiotics, exert positive effects on the composition of the gut microflora [3]. Every modification of the microbiome loops back to influence the health and metabolic status of the host. In this manner, the host and microbiome are interconnected in infinite symbiosis. On the host’s side, these inherent mechanisms are controlled by mucous tissue. For this reason, focus on the mucosa may help explain the mechanisms that follow microbiota modulation.

Intestinal mucosa is comprised of the epithelium, lamina propria, and smooth muscle. The outermost layer of the small intestine is lined with absorptive, columnar epithelial cells (enterocytes) with interspersed goblet cells that secrete mucins, and Paneth cells that secrete antimicrobials [4]. The mucosa forms intestinal villi and crypts, which take part in nutrient absorption and cellular turnover. Avian GIT reflects adaptation to type of food, environment, and motility [5]. The digestive system has to be flexible to accommodate a wide range of diets over the life cycle. Aside from digestion and absorption of ingested food, GIT plays a role in protecting internal tissues from luminal content. Such a barrier is physical, immunological, or microbial [6].

The immunological barrier of the intestine is formed by gut-associated lymphoid tissue (GALT), which includes bursa of Fabricius, caecal tonsils (double), Peyer’s patches (up to five), lymphoid aggregates (mostly in hindgut—urodeum and proctodeum) and intraepithelial T lymphocytes [7]. Innate and adaptive barrier mechanisms in the gut were reviewed by Kelsall (2008) [8]. Innate mechanisms include pattern recognition receptors (e.g., TLR, Toll-like receptors), mucins, cytokines and host defence peptides (HDP). Adaptive mechanisms develop in submucosal lamina propria, which is rich in all kinds of lymphatic cells, including dendritic cells, macrophages, and B lymphocytes. The latter produce secretory IgA that are released into lumen and neutralise antigens locally, without activation of inflammatory responses or the complement system. The immunological mechanisms are based on the subtle balance between immune tolerance and immune responses.

In avian species, GIT is relatively short, which results in the fast transfer time of chyme through the intestines [5]. It is much harder for the bacteria to colonise the proximal segments of the intestines. This is why most bacteria populations colonise crop, distal ileum, and caeca, where peristaltic movement is slower [9]. Stanley et al. (2014) reviewed the literature on spatial microbiome composition along the chicken GIT [10]. Currently, the most integrated report on chicken microbiome composition has been published by Wei et al. [11]. Based on 16s rRNA sequences from Sanger sequencing, chicken guts contain 70% *Firmicutes* (including 8% *Lactobacillus*), 12.3% *Bacteroidetes*, and 9.3% *Proteobacteria*. *Bifidobacterium* from *Actinobacteria* phyla was represented by about 1% of the sequences. The microbiome composition is strongly influenced by environmental rather than genetic factors [12]. Prebiotics, which are defined non-digestible oligosaccharides, are potent modulators of the intestinal microflora. Dietary prebiotics are metabolized only by particular intestinal bacteria, which in turn selectively stimulates their growth. The most common prebiotics include inulin, fructooligosaccharides (FOS), galactooligosaccharides (GOS), or mannanoligosaccharides (MOS) followed by novel prebiotics, such as xylooligosaacharides or isomaltooligosaccharides. For the detailed information on application of prebiotics in poultry we reference to some recent review papers [13–15].

Recent reports emphasize that early-life microbial stimulation has long-term effects on intestinal development and health by promoting maturation of the immune system and development of oral tolerance [16–18]. It also has been shown that microflora disturbances early in life by antimicrobials has long-lasting negative effects on the animal [19, 20]. For this reason, *in ovo* technologies have been adapted to administer a bioactive compound such as prebiotic, probiotic or synbiotic during embryonic development. *In ovo* technologies apply to early-stage (e.g., day 12 of egg incubation) or late-stage (e.g., day 17–18 of egg incubation) chicken embryos, reviewed by Roto et al. (2016) [21]. These methods are referred to by their authors as *in ovo* stimulation (early-stage embryos) [22] or *in ovo* feeding (late-stage embryos) [23–25]. We report effects of *in ovo* stimulation of the chicken microflora. This method is based on *in ovo* delivery of prebiotics or synbiotics on day 12 of egg incubation [26]. At this time-point, the chorioallantoic membrane is highly vascularized, which allows for a passive transfer of the small-weight oligosaccharides from the air cell to the blood vessels surrounding the embryo. The purpose of the early delivery of prebiotics is to boost growth of the indigenous microflora present in the egg, so that the microbiome can be developed along with the embryo. The *in ovo* stimulation has been patented [27] and validated at laboratory scale and in field trials [28–30].

Among many bioactive compounds tested for *in ovo* stimulation, GOS prebiotic demonstrated particularly beneficial properties in chickens, including an increase in the number of lactobacilli and bifidobacteria at hatching [28], long-term transcriptional modulation of the host [31], microstructure of the small intestine [32], immune system development [33–35], and improvement in broiler performance [30]. GOS was also used in chickens for dietary interventions to improve performance traits [36] as well as resistance against heat stress [37] and *Salmonella* colonisation [38, 39]. The effects of GOS delivered *in ovo* on the chicken were evaluated on multiple levels. The first sets of traits analysed in meat-type chickens was performance, measured by growth, efficiency of nutrition, and meat quality [29, 40]. A further step of looking into the effects of *in ovo* stimulation was the examining the development of intestinal tissue and microflora [41]. Analysis of morphology was followed by physiological modulation of nutrients and hormones [42]. Finally, the effects of GOS delivered *in ovo* on gene expression in spleen or caecal tonsils was demonstrated [31]. The next step was to study the effects of the molecular interaction between host and microbiome at their actual interface, which is intestinal mucosa.

This paper aims to provide insights into the mechanisms that drive beneficial effects of GOS stimulation *in ovo* in chicken. It focuses on both sides of microbiota-host interaction, i.e., selected microbial populations and intestinal mucosa physiology. For this purpose, the following effects of GOS delivered *in ovo* analyzed was analysed: (1) abundance of *Bifidobacterium* and *Lactobacillus* in the intestinal chyme as well as (2) the immune and physiological parameters of the intestinal mucosa measured by gene expression.

## Materials and methods

### GOS *in ovo* delivery

Prebiotic GOS was used for *in ovo* stimulation by *in ovo* delivery on day 12 of egg incubation. The GOS used in this study (trade name: Bi^2^tos, Clasado Biosciences Ltd., Jersey, UK) is manufactured by enzymatic transgalactosylation of the milk lactose by the whole cells of *Bifidobacterium bifidum* 41171 [43]. The GOS product obtained this way is a dry powder containing a mixture (wt:wt) of the following oligosaccharides: 45% lactose, 9.9% disaccharides [Gal (β 1–3)-Glc; Gal (β 1–3)- Gal; Gal (β 1–6)- Gal; Gal (α 1–6)- Gal], 23.1% trisaccharides [Gal (β 1–6)-Gal (β 1–4)- Glc; Gal (β 1–3)- Gal (β 1–4)- Glc], 11.55% tetrasaccharides [Gal (β 1–6)- Gal (β 1–6)- Gal (β 1–4)- Glc], and 10.45% pentasaccharides [Gal (β 1–6)- Gal (β 1–6)- Gal (β 1–6)- Gal (β 1–4)- Glc]. Injection dose was aseptically prepared by dissolving 3.5 mg of GOS mixture in 0.2 ml of physiological saline. The GOS/saline solution was manually deposited into an air chamber with a syringe and a needle. The *in ovo* injection was done in two steps: (1) the whole was punctured in the blunt end of the egg containing viable, 12-day-old embryo and (2) the 0.2 ml of GOS/saline solution was injected into the air chamber. The size of the needle used for puncturing the hole (0.9 mm) was bigger than the needle used for *in ovo* injection (0.45 mm). This way, enough space was left to let go air that was replaced by the solution. Also, only the outer egg membrane was punctured prior to injection. Precaution was taken not to break the semi-permeable inner egg membrane, which at this point (i.e., day 12 of eggs incubation) is highly vascularized and moist. Using blue dye for tracking, we determined that the substance (i.e., GOS) injected this way is gradually diffused through the inner egg membrane into the bloodstream of the embryo [22]. At the last step of the *in ovo* procedure, the hole punctured in the egg is sealed with a food-safe glue, which protects from water loss from the embryo. The egg tray is returned to the incubator within 20 minutes.

### Animal trial

The trial was conducted on Ross 308 broiler chickens. Fertilized eggs (400 eggs) were incubated in standard conditions. On day 12 of incubation, the eggs were candled and viable embryos received an *in ovo* injection that contained either a biologically active compound (prebiotic) or physiological saline (mock injection). Experimental eggs (200 eggs) were injected *in ovo* with GOS. Control eggs (200 eggs) were mock-injected *in ovo* with 0.2 ml of physiological saline. All handling procedures of the eggs (punching hole, injecting liquid, sealing) were the same for GOS-injected and mock-injected eggs. After *in ovo* injection, incubation continued until hatching. One-day-old chicks were transported to the farm and distributed into pens (4 replicate pens/group, 10 birds/pen). The animals were reared in an open system, on a wooden litter with *ad libitum* access to feed and water. **Table 1.** shows the diet composition. On day 42 post-hatching randomly selected chickens (n = 10) were sacrificed by cervical dislocation and the intestinal samples were taken. The study was approved by the Local Ethics Committee for Animal Research (http://lke.utp.edu.pl) located at the Faculty of Animal Breeding and Biology, UTP University of Science and Technology in Bydgoszcz (study approval reference number 16/2014).

**Table 1:** Diet composition and nutritional value of the feed used for animal trial.

	Feeding phase	
**Ingredients (%)**	**Starter (1–21 days)**	**Grower (21–42 days)**
Corn	22	31.6
Wheat	19.5	15
Soybean meal	31.5	25
Wheat middlings	13	15
Corn gluten	10	10
Soybean oil	1.32	1.1
Calcium Carbonate	1.2	1
Dicalcium Phosphate	0.5	0.5
NaCl	0.2	0.2
Sodium bicarbonate	0.1	0.1
Vitamin-mineral premix 1 ^1^	0.3	-
Vitamin-mineral premix 2 ^2^	-	0.3
Phytase	0.1	0.1
Coccidiostat	0.1	0
Color additives	0.1	0.1
Methionine	0.08	-
**Calculated nutritional value of the diet (%)**		
Protein	24.00	21.00
Lipid	4.50	4.50
Crude fiber	4.50	4.00
Ash	7.00	6.00
Lysine, %	1.10	1,00
Methionine, %	0.35	0.30
Calcium, %	1.30	1.10
Available P, %	0.70	0.60
Sodium, %	0.15	0.20

^1^Supplied per kilogram of diet: vitamin A, 12,000 IU; vitamin D3, 3,600 IU; vitamin E, 50.1 mg; vitamin B1, 3 mg; vitamin B12, 0.04 mg; vitamin B2, 6 mg; vitamin B6, 3.99 mg; CuSO4 5H2O (Cu, 10mg), 38.26mg; Ca(IO3)2 (I, 1.50mg), 2.31mg; FeCO3 (Fe, 45mg), 93.15mg; MnO (Mn, 36mg), 46.44mg; MnSO4 (Mn, 35mg),110.88mg; Na2SeO3 (Se, 0mg), 0.43mg; ZnO (Zn, 51mg), 63.24mg.

^2^Supplied per kilogram of diet: vitamin A, 10,000 IU; vitamin D3, 3,000 IU; vitamin E, 41.68 mg; vitamin B1, 2.90 mg; vitamin B12, 0.03 mg; vitamin B2, 5 mg; vitamin B6, 3.33 mg; CuSO4 5H2O (Cu, 8mg), 32.72mg; Ca(IO3)2 (I, 1.25mg), 1.93mg; Fe2O3 (Fe, 560mg), 800.8mg; FeCO3 (Fe, 38mg), 77.63mg; MnO (Mn, 30mg), 38.70mg; MnSO4 (Mn, 30mg), 92.40mg; Na2SeO3 (Se, 0mg), 0.36mg; ZnO (Zn, 43mg), 52.7mg.

During sampling the intestinal tract was excised and separated into four gut segments (duodenum, jejunum, ileum, and caecum). Each gut segment used for sampling contained approximately 5-cm-long fragment that was cut from the same sites in all animals. The duodenum was sampled in the middle of the duodenal loop (mid-duodenum). The jejunal segment was cut about 30–35 cm proximally from Meckel’s diverticulum (mid-jejunum). The ileal segment (distal ileum) was cut about 10–15 cm proximally to caeca. The caecum was sampled distal to caecum tonsil. Intestinal content was removed from each gut segment separately and frozen (-80°C) prior to bacterial DNA isolation. The gut segments were then rinsed in PBS and the mucosal layer was scraped with a glass slide. Mucosal scrapings were preserved in a DNA/RNA Stabilization Reagent (Fisher Molecular Biology, Terry Drive, PA, USA) prior to RNA isolation.

### RNA and DNA isolation

Total bacterial DNA was isolated from the intestinal content. Approximately 100 mg of material was lysed and purified using a Genomic Mini AX Stool (A&A Biotechnology, Gdynia, Poland), according to manufacturer’s instruction. Next, isolated DNA was evaluated using the NanoDrop 2000 (Thermo Scientific Nanodrop Products, Wilmington, USA) and agarose gel electrophoresis. The evaluated DNA was diluted to a working concentration of 2 ng/μl and stored at 4°C prior to further analyses.

Total RNA was isolated from the mucosal scrapings sampled from the chicken intestines. About 100 mg of the mucosal scrapings were first homogenized in 1 ml of Trizol (Invitrogen, Carlsbad, USA) using a TissueRuptor homogenizer (Qiagen GmbH, Hilden, Germany). RNA was purified from the lysate using a Universal RNA Purification Kit (EURx, Gdańsk, Poland), according to manufacturer’s instruction. All RNA samples were evaluated using the NanoDrop 2000 (Thermo Scientific Nanodrop Products, Wilmington, USA) and agarose gel electrophoresis. Additionally, 10% of the RNA samples were evaluated on an Agilent 2100 Bioanalyzer using an Agilent RNA 6000 Nano Kit (Agilent Technologies, Santa Clara, CA, USA). RNA isolates were kept frozen at -20°C.

### Bacteria quantification with qPCR

The relative abundances of *Bifidobacterium* spp. and *Lactobacillus* spp. in chyme samples from the duodenum, jejunum, ileum, and caecum were determined using quantitative PCR (qPCR) carried out on a LightCycler 480 II System (Roche-Diagnostics, Basel, Switzerland). A total reaction volume of 10 μl in a 384-well plate format contained 5 μl Maxima SYBR Green qPCR Master Mix (Thermo Scientific/Fermentas, Vilnius, Lithuania), 0.2 μM of each primer, specific to 16s rDNA of *Bifidobacterium* spp. (F: GCGTGCTTAACACATGCAAGTC, R: CACCCGTTTCCAGGAGCTATT) [44], *Lactobacillus* spp. (F: AGCAGTAGGGAATCTTCCA, R: CACCGCTACACATGGAG) [45, 46] or universal bacteria (F: ACTCCTACGGGAGGCAGCAGT, R: GTATTACCGCGGCTGCTGGCAC) [47] and 2 ng of bacterial DNA template. Each reaction was performed in four technical replicates. Thermal cycling consisted of initial denaturation at 95°C for 5 min, followed by 40 cycles of amplification: denaturation at 95°C for 15s, annealing at 58°C for 15s, and elongation at 72°C for 45s. Fluorescence was measured at the end of each extension step. After amplification, a melting curve was generated by increasing the temperature in small increments to 98°C and measuring the fluorescence of melting amplicons. Average Ct values of the four technical replicates obtained from the LightCycler 480 II System software were used for data analysis. Single Ct values differing by more than 0.3 were considered outliers. PCR efficiency for each primer pair was calculated in the LightCycler 480 II software based on the separate reaction of 5 dilutions (1x, 0.5x, 0.25x, 0.125x and 0.0625x) of pooled bacterial DNA template.

The relative abundances of the bacteria in the chyme were calculated as follows:

Relative Abundances [%] = (E universal)^Ct universal^ / (E target)^Ct target^

Where, E universal is the efficiency of qPCR with primers for all bacteria, Ct universal is the Ct values for reaction with primers for all bacteria, E target is the efficiency of qPCR with primers specific for *Bifidobacterium* spp. or *Lactobacillus* spp., and Ct target is the Ct values for reaction with primers for *Bifidobacterium* spp. or *Lactobacillus* spp. [48].

### Gene expression in intestinal mucosa

Gene expression analysis in the intestinal mucosa of chickens stimulated *in ovo* with GOS was performed for two gene panels. The full list and function of the genes are presented in **Table 2**. Panel (1) included genes related to intestinal innate immune responses, such as interleukins (*IL-1β*, *IL-10* and *IL-12p40*) and HDP, defensins (*AvBD1*) and cathelicidins (*CATHL2*). Panel (2) contained genes involved in intestinal barrier function and nutrient sensing, including mucous compound (*MUC6*), tight junctions (TJ) proteins (*CLDN1* and *TJAP1*), free fatty acids (FFA) receptors (*FFAR2* and *FFAR4*) and glucose transporters (*GLUT1*, *GLUT2* and *GLUT5*). Normalization of the expression levels of the target genes was performed with the geometric mean of two housekeeping genes: glucose-6-phosphate dehydrogenase (*G6PDH*) and beta actin (*ACTB*) [49]. Gene expression analysis was performed with two step reverse transcription quantitative PCR (RT-qPCR).

**Table 2:** Molecular function of the intestinal genes primer sequences used for RT-qPCR.

Gene	Name	NCBI gene ID	Function in intestinal mucosa^1^	Primer sequences ^2^ (5’ → 3’)	Ref
**Panel 1. Cytokine genes**					
***IL-1β***	Interleukin 1 beta	395196	Pro-inflammatory cytokine produced by activated macrophages	F: GGAGGTTTTTGAGCCCGTC R: TCGAAGATGTCGAAGGACTG	this study
***IL-10***	Interleukin 10	428264	Anti-inflammatory cytokine, immunoregulator in the intestinal tract	F: CATGCTGCTGGGCCTGAA R: CGTCTCCTTGATCTGCTTGATG	[53]
***IL-12p40***	Interleukin 12 subunit beta	404671	Cytokine with broad biological activities, activates T and NK cells, stimulates long-term immune responses	F: TTGCCGAAGAGCACCAGCCG R: CGGTGTGCTCCAGGTCTTGGG	[54]
**Panel 2. Host defence peptide (HDP) genes**					
***AvBD1***	Avian beta-defensin 1	395841	Host defence peptide involved in resistance of epithelia to microbial colonization	F: AAACCATTGTCAGCCCTGTG R: TTCCTAGAGCCTGGGAGGAT	this study
***CATHL2***	Cathelicidin 2	420407	Host defence peptide, inflammatory response regulator, functions in chemotaxis	F: AGGAGAATGGGGTCATCAGG R: GGATCTTTCTCAGGAAGCGG	this study
**Panel 3. Barrier function genes**					
***MUC6***	Mucin 6	414878	Forms insoluble mucin barrier that protects gut lumen and modulates mucus composition	F: TTCAACATTCAGTTCCGCCG R: TTGATGACACCGACACTCCT	this study
***CLDN1***	Claudin 1	424910	Component of tight junctions, regulates permeability of epithelia and water homeostasis	F: TCTTCATCATTGCAGGTCTGTC R: AACGGGTGTGAAAGGGTCAT	this study
***TJAP1***	Tight junction associated protein 1	421455	Component of tight junctions, takes part in vesicular trafficking	F: AGGAAGCGATGAATCCCTGTT R: TCACTCAGATGCCAGATCCAA	this study
**Panel 4. Nutrient sensing genes**					
***FFAR2***	Free fatty acid receptor 2	100859369	Receptor for short chain free fatty acids, regulates energy homeostasis through adipogenesis and controls intestinal immunity	F: GCTCGACCCCTTCATCTTCT R: ACACATTGTGCCCCGAATTG	this study
***FFAR4***	Free fatty acid receptor 4	428963	Receptor for medium and long chain free fatty acids (e.g., omega-3), represses macrophage-induced tissue inflammation	F: AGTGTCACTGGTGAGGAGATT R: ACAGCAACAGCATAGGTCAC	this study
***GLUT1***	Glucose transporter 1	396130	Basal, constitutive glucose transporter with broad substrate sensitivity	F:AGATGACAGCTCGCCTGATG R:GTCTTCAATCACCTTCTGCGG	this study
***GLUT2***	Glucose transporter 2	396272	Na(+)/glucose cotransporter in the small intestine, glucose sensor	F:GGAGAAGCACCTCACAGGAA R:CAGGCTGTAACCGTACTGGA	this study
***GLUT5***	Glucose transporter 5	419438	Fructose uptake by small intestine	F:ACGGTTCCCAGAGCAAGTTA R:GTCTTGCATGTATGGGGCTG	this study
**5. Reference genes**					
***ACTB***	Actin, beta	396526	Ubiquitous cytoskeletal actin involved in cell motility, structure, integrity and intercellular signalingsignalling	F: CACAGATCATGTTTGAGACCTT R: CATCACAATACCAGTGGTACG	[49]
***G6PDH***	Glucose-6-Phosphate Dehydrogenase	428188	Cytosolic enzyme that produces NADPH in reductive biosynthetic reactions	F: CGGGAACCAAATGCACTTCGT R: GGCTGCCGTAGAGGTATGGGA	[49]

^1^ gene function derived from GeneCards (http://www.genecards.org)

^2^ F–Forward primer, R–Reverse primer

For gene expression analysis, 1250 ng of total RNA of each sample was reversely transcribed to cDNA using Maxima First Strand cDNA Synthesis kit (Thermo Scientific/Fermentas, Vilnius, Lithuania). RT was performed at the volume of 10 μl, including 2 μl of 5x Reaction Mix and 1 μl of Maxima Enzyme Mix. Next, qPCR reactions were prepared at 10μL total volume in a 384-well plate format and contained: 5μl of Maxima SYBR Green qPCR Master Mix (Thermo Scientific/Fermentas, Vilnius, Lithuania), 1μM of forward and reverse primers, and 140 ng of cDNA. Oligonucleotide primers (**Table 1**) were designed using an NCBI/Primer-BLAST [50]. If it was possible, amplicon sequences spanned exon-exon junctions. RT-qPCR reactions were performed in the LightCycler 480 System (Roche-Diagnostics, Basel, Switzerland) and consisted of: initial denaturation at 95°C for 15 min, 40 cycles of amplification (denaturation at 95°C for 15s, annealing at 58°C for 20s, and elongation at 72°C for 20s) and melting curve. The annealing temperature was 58°C for all target genes except *IL12p40*, for which it was 65°C. Fluorescence was measured at the end of each elongation step. The melting curve was generated by increasing the temperature in small increments up to 98°C and measuring the fluorescence of the melting amplicon.

Relative gene expression was calculated with ^ΔΔ^Ct algorithm and the amount of the target gene was calculated with formula 2^–ΔΔCt^ [51]. A Multiexperiment Viewer (**MeV**) version 4.9 [52] was used to create a Hierarchical Cluster Tree based on fold change values. After loading data, results were clustered using the Hierarchical Clustering function with the standard option (Pearson Correlation) to prepare a heat map.

### Statistical analysis

Statistical analysis was conducted with the SAS statistical software version 9.4 (SAS Institute, Cary, NC, USA). In both datasets (microbial communities and intestinal gene expression), the significance of the effects and their interaction was analysed with two-way ANOVA. The effects were: *in ovo* injected group (GOS vs. C) and intestinal segment (duodenum, jejunum, ileum, and caecum). An HSD Tukey post hoc test was used to determine differences in gene expression. Significance thresholds *P* < 0.05, 0.01 and 0.001 were used.

## Results

### Relative abundance of *Lactobacillus* spp. and *Bifidobacterium* spp.

**Fig 1:**
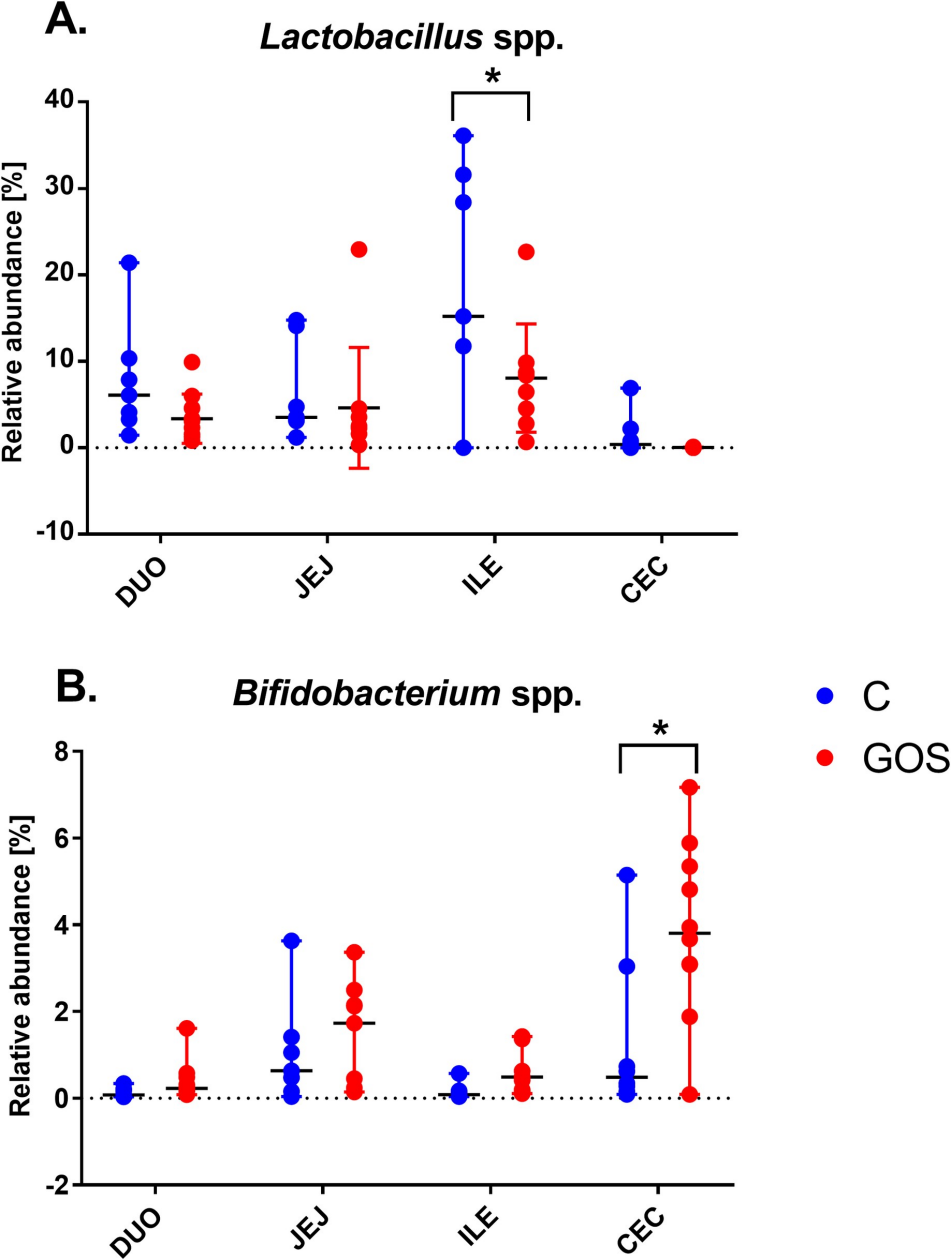
The relative abundance of *Bifidobacterium* spp. (A) and *Lactobacillus* spp. (B) in chyme from different sections of GIT in chickens supplemented *in ovo* with GOS prebiotic. GOS was delivered *in ovo* on day 12 of egg incubation. Samples of intestinal content were collected from 42-days-old chickens. Intestinal content was sampled from four distinct intestinal segments (duodenum, jejunum, ileum, and caecum) (n = 10). Bacteria quantification was done with qPCR based on bacterial DNA isolated from chyme. Statistical analysis was performed with two-way ANOVA with a Tukey HSD post hoc test. Significant differences found at *P* < 0.05.

All the raw data obtained in this study have been made available in S1 File. Intestinal segment (*P* < 0.001) and prebiotic treatment *in ovo* (*P* < 0.01) had a significant effect on the relative abundance of microbial communities (*Bifidobacterium* and *Lactobacillus*) in the chyme. In *Bifidobacterium* spp. we found significant interaction between the intestinal section and prebiotic supplementation (*P* < 0.05). **Fig 1** presents the relative abundance of (A) *Bifidobacterium* spp. and (B) *Lactobacillus* spp. in four sections of intestinal content (duodenum, jejunum, ileum, and caecum). Average values of *Bifidobacterium* ranged from 0.03% (in ileum) to 1.3% (in caecum) of C and from 0.4% (in ileum) to 3.9% (in caecum) of the GOS-injected group. The highest relative abundance of *Bifidobacterium* spp. (~4%) was found in caecum of GOS. It was significantly higher than caecum of C (*P* < 0.001) but also significantly higher than in other intestinal segments (*P* < 0.05).

There were more pronounced differences between treatment groups and intestinal section for *Lactobacillus* spp. There were higher values of *Lactobacillus* abundance in C vs. GOS. Median values of *Lactobacillus* spp. ranged from 0.40% (in caecum) to 15.20% (in ileum) of C and from 0.02% (in caecum) to 8.36% (in ileum) of GOS. GOS decreased *Lactobacillus* spp. along all GIT. The largest lactobacilli populations in this trial were found in ileum of C (*P* < 0.05).

### Intestinal gene expression

#### Hierarchical clustering of gene expression profiles

**Fig 2:**
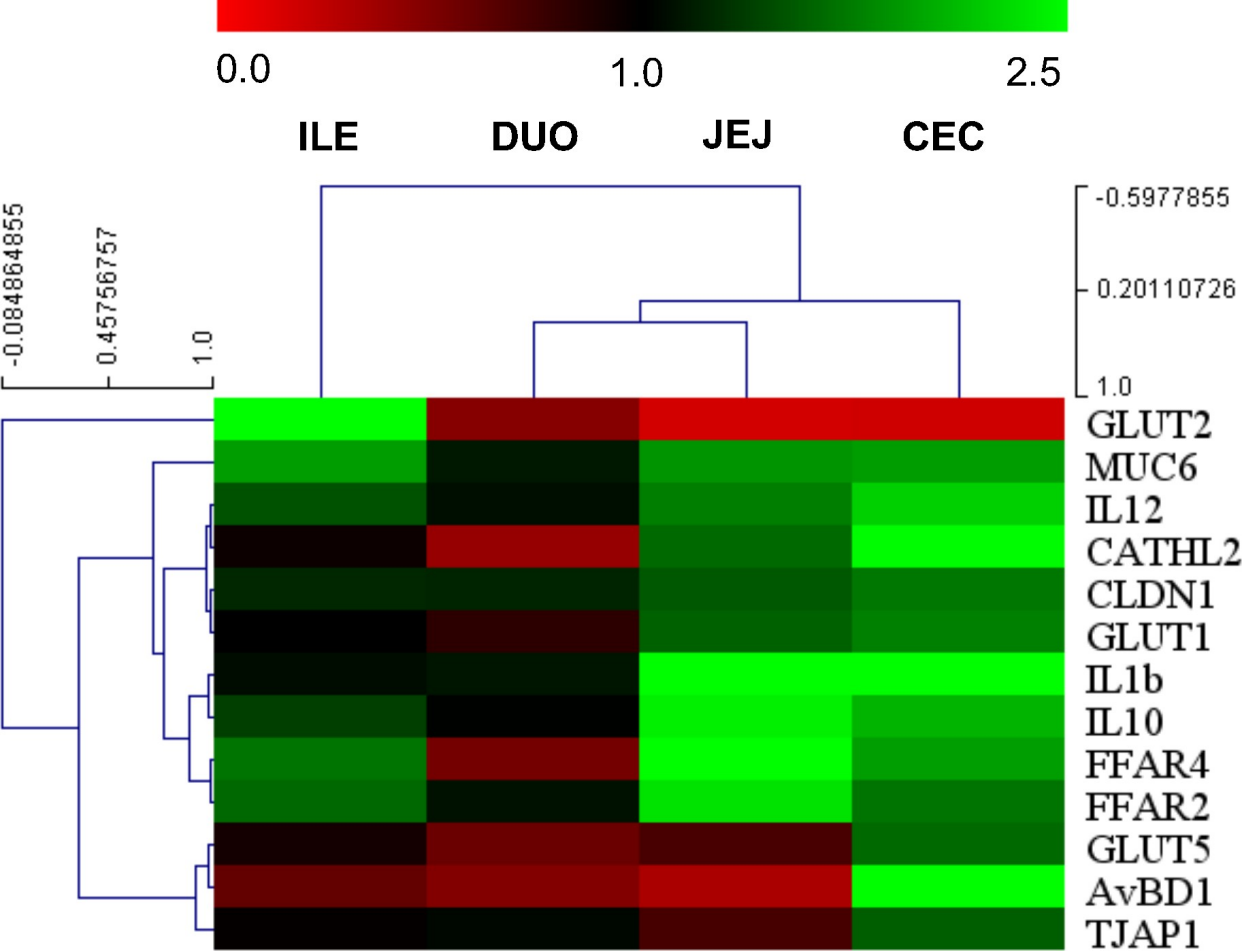
A heat map of hierarchically clustered gene expression in different segments of intestinal mucosa in chicken treated with GOS *in ovo*. Intestinal segments: DUO–duodenum, JEJ–jejunum, ILE–ileum, and CEC–caecum. In ovo injection was carried out on day 12 of egg incubation. Intestinal samples (n = 10) collected from chickens on day 42 post-hatching. RT-qPCR data were generated with custom-designed primers used for amplification with SYBR green dye; Glucose-6-phosphate dehydrogenase (G6PDH) and beta-actin (ACTB) were used as reference genes; relative gene expression (fold change) calculated as 2^–ΔΔCt^. A Multiexperiment Viewer version 4.9 (MeV) was used for constructing a Hierarchical Cluster Tree based on fold change. Colours (red-black-green) show relative gene expression changes in GOS vs. C (red: down-regulated, green: up-regulated genes).

Hierarchical gene and sample clustering allowed determining the relationships between the samples and co-regulated genes. Based on correlation data of intestinal gene expression in GOS vs. C (Fig 2), we have determined that the pattern of mRNA expression was similar in jejunum and caecum. Three major gene clusters were identified, consisting of (1) *IL-12p40*, *CATHL2*, *CLDN1* and *GLUT1*; (2) *IL-1β*, *IL-10*, *FFAR2* and *FFAR4*; and (3) *GLUT5*, *AvBD1* and *TJAP1*.

#### Effects of in ovo treatment and intestinal segment on gene expression

Effects of the *in ovo* treatment group and intestinal segment on mRNA gene expression in chicken intestinal mucosa were included in ANOVA (**Table 3**). The expression of every gene analysed depended on either variable (*P* < 0.05 or lower). Interaction was found significant for *IL-1β*, *TJAP1*, *FFAR4*, *GLUT1*, and *GLUT2* (*P* < 0.05 or lower).

**Table 3:** Effects of experimental group, intestinal segment and their interaction on mRNA expression of immune, barrier function and nutrient sensing genes in chicken intestinal mucosa.

Gene	Treatment ^1^	Intestine ^2^	Treatment x Intestine ^3^
**Panel 1. Cytokine genes**			
***IL-1β***	< 0.001	< 0.001	< 0.01
***IL-10***	< 0.001	< 0.01	ns
***IL-12p40***	< 0.001	< 0.001	ns
**Panel 2. Host defence peptide (HDP) genes**			
***AvBD1***	ns	<0.001	ns
***CATHL2***	ns	<0.01	ns
**Panel 3. Barrier function genes**			
***MUC6***	< 0.01	< 0.001	ns
***CLDN1***	< 0.01	< 0.001	ns
***TJAP1***	Ns	< 0.001	<0.05
**Panel 4. Nutrient sensing genes**			
***FFAR2***	< 0.001	ns	ns
***FFAR4***	< 0.05	< 0.001	< 0.01
***GLUT1***	< 0.05	< 0.001	< 0.05
***GLUT2***	ns	< 0.001	< 0.01
***GLUT5***	ns	< 0.001	ns

Effects

^1^ *in ovo* delivery of GOS prebiotic (GOS vs C)

^2^ intestinal segment (duodenum, jejunum, ileum, or caecum) from which a chyme sample was collected and

^3^ the interaction between *in ovo* treatment and the intestinal segment on mRNA expression of immune and physiological genes in chicken mucosa. Gene expression analysis done with RT-qPCR. Significance of effects calculated with two-way ANOVA. Significance levels: *P* < 0.05, 0.01 or 0.001.

#### Relative expression of intestinal immune-related genes

**Fig 3:**
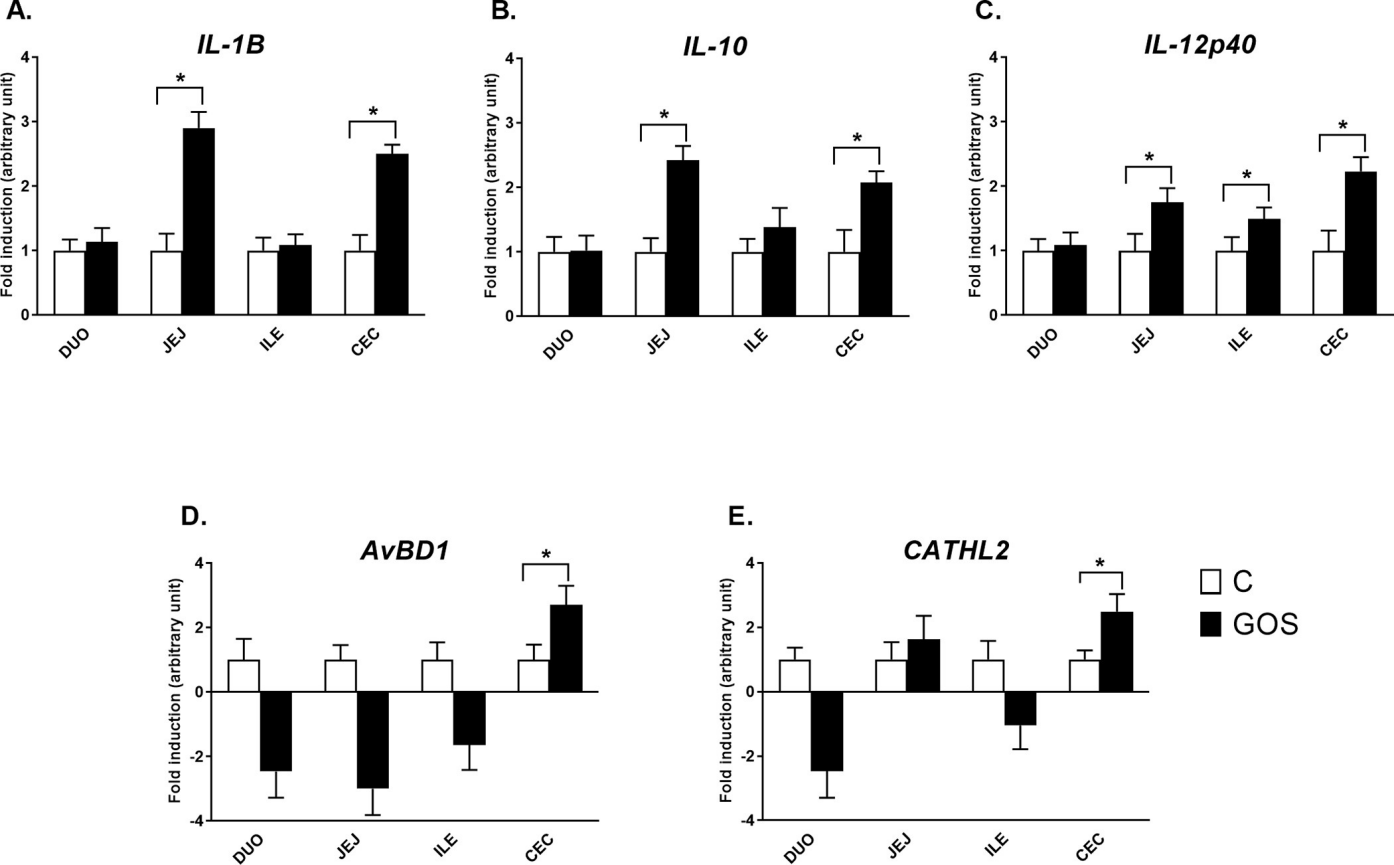
Relative mRNA expression of intestinal immune-related genes in different segments of intestinal mucosa in chickens injected *in ovo* with GOS. The panel includes genes encoding cytokines, (A) *IL-1β*, (B) *IL10* and (C) *IL12p40*; and host defence peptides, (D) *AvBD1* and (E) *CATHL*. Intestinal segments: DUO–duodenum, JEJ–jejunum, ILE–ileum, and CEC–caecum. *In ovo* treatment groups: control (white bars), injected *in ovo* with physiological saline; GOS (black bars)–injected *in ovo* with galactooligosaccharides. *In ovo* injection was carried out on day 12 of egg incubation. Intestinal samples (n = 10) were collected from chickens on day 42 post-hatching. RT-qPCR data were generated with custom-designed primers used for amplification with SYBR green dye; Glucose-6-phosphate dehydrogenase (*G6PDH*) and beta-actin (*ACTB*) were used as reference genes; relative gene expression calculated as 2^–ΔΔCt;^ Two-way ANOVA with post hoc HSD Tukey test was used to compare the groups. Asterisk indicates pair-wise significant differences (*P* < 0.05). The results of fold induction less than 1 have been transformed by the formula -1/fold induction.

The relative expression of the intestinal innate immune response genes presented two patterns of gene expression regulation (**Fig 3**). Cytokine genes (*IL-1β*, *IL-10* and *IL-12p40*) were up-regulated in jejunum and caecum of GOS (*P* < 0.05). The expression profile of those cytokines in duodenum and ileum did not deviate much from C, except *IL-12p40*, which was slightly up-regulated also in ileum (*P* < 0.05). HDP genes (*AvBD1* and *CATHL*) were significantly up-regulated only in caecum of GOS (*P* < 0.05). In other intestinal segments (duodenum, jejunum, and ileum), those genes were down-regulated in GOS, but beyond statistical significance (*P* > 0.05). The fold change of the gene expression of significantly up-regulated immune genes in caecum ranged between 2.07 (*IL-10*) and 2.70 (*AvBD1*).

#### Relative expression of barrier function and nutrient sensing genes

**Fig 4:**
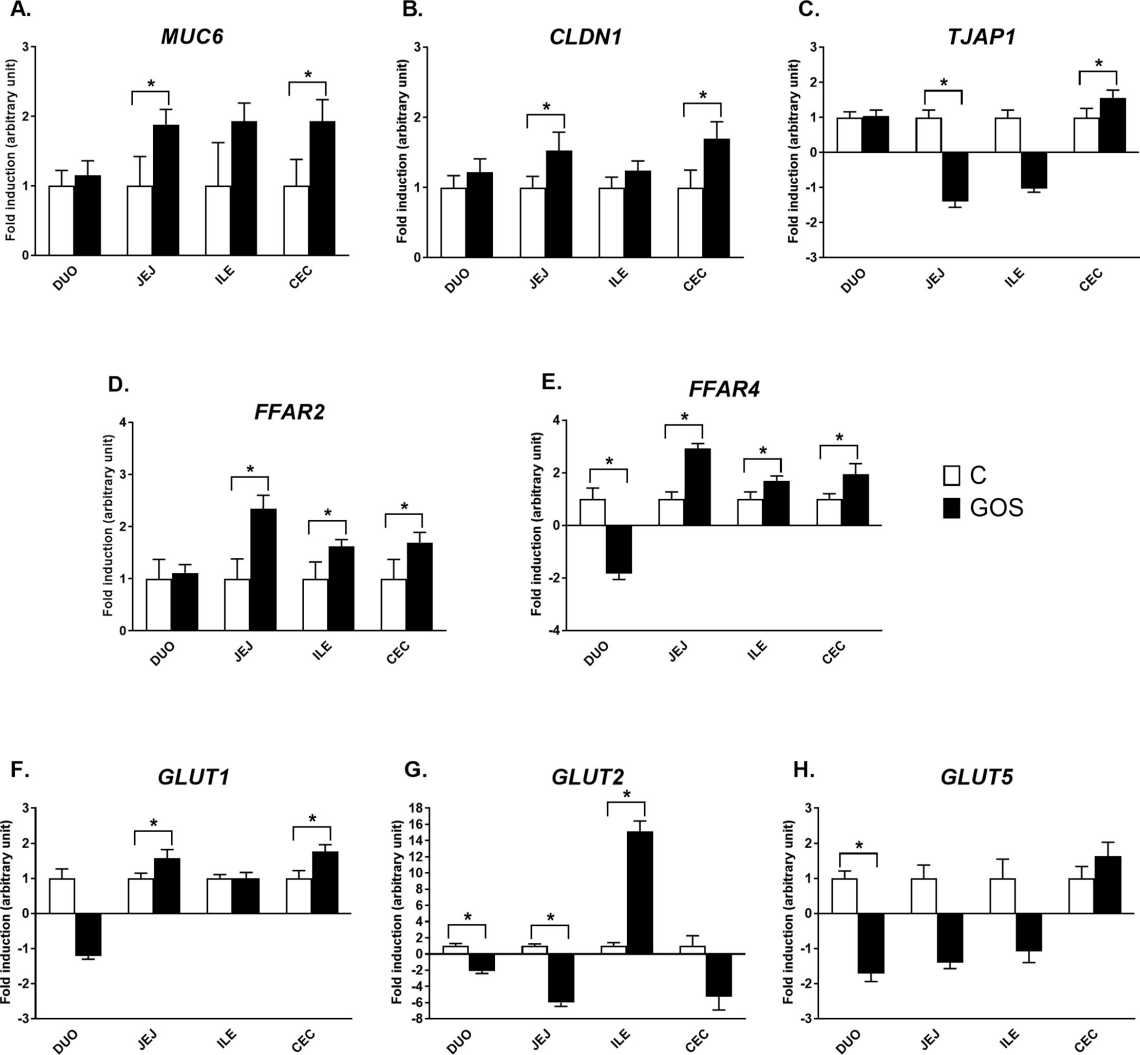
Relative mRNA expression of barrier function and nutrient sensing genes in different segments of intestinal mucosa in chickens injected *in ovo* with GOS. The panel includes genes encoding mucin, (A) *MUC6*; tight junctions components, (B) *CLDN1* and (C) *TJAP1*; free fatty acid receptors, (D) *FFAR2* and (E) *FFAR4*; and glucose transporters, (F) *GLUT1*, (G) *GLUT2* and (H) *GLUT5*. Intestinal segments: DUO–duodenum, JEJ–jejunum, ILE–ileum and CEC–caecum. *In ovo* treatment groups: control (white bars)–injected *in ovo* with physiological saline; GOS (black bars)–injected *in ovo with* galactooligosaccharides. *In ovo* injection was carried out on day 12 of egg incubation. Intestinal samples (n = 10) were collected from chickens on day 42 post-hatching. RT-qPCR data were generated with custom-designed primers used for amplification with SYBR green dye; Glucose-6-phosphate dehydrogenase (*G6PDH*) and beta-actin (*ACTB*) were used as reference genes; relative gene expression calculated as 2^–ΔΔCt;^ Two-way ANOVA with a post hoc HSD Tukey test was used to compare the groups. Asterisk indicates pair-wise significant differences (*P* < 0.05). The results of fold induction less than 1 have been transformed by the formula -1/fold induction.

The mRNA expression of barrier function and nutrient sensing was varied and dependent mostly on intestinal segment (**Fig 4**). *MUC6* and *CLDN1* followed a similar pattern of gene expression regulation, with moderate (FC ~1.5–2.0) but significant up-regulation in jejunum and caecum of GOS (*P* < 0.05). *TJAP1* had a distinct pattern of mRNA expression, also with moderate but significant down-regulation in jejunum (FC -1.4, *P* < 0.05) and up-regulation in caecum (FC 1.6, *P* < 0.05) of GOS. Free fatty acid receptors (*FFAR2* and *FFAR4*) were up-regulated in jejunum, ileum, and caecum of GOS (*P* < 0.05). *FFAR4* was significantly down-regulated in duodenum of GOS (*P* < 0.05). The most pronounced changes were determined in *GLUT2*, which was up-regulated in ileum of GOS (FC 15.2, *P* < 0.05), but down-regulated in duodenum (FC -2.1, *P* < 0.05), jejunum (FC -6, *P* < 0.05) and (suggestively) in caecum of GOS (FC -5.3, *P* > 0.05). *GLUT1* was up-regulated in jejunum and caecum of GOS (*P* > 0.05), which seemed like a general pattern of expression regulation dependent on *in ovo* treatment. *GLUT5* was significantly down-regulated only in duodenum of GOS (*P* < 0.05), with a slight but not significant fluctuation in mRNA expression in other intestinal segments.

## Discussion

We have evaluated the impact of *in ovo* stimulation of chicken embryos with GOS prebiotic on the development of microbial populations along the GIT as well as the modulatory role in mucosal gene expression. We have demonstrated that a single injection of GOS on day 12 of egg incubation can efficiently and stably promote the growth of certain species of indigenous microflora, which has life-long consequences for the intestinal environment of broiler chickens. As such, these data provide insight into the spatial and functional interaction at the interface between intestinal microflora and mucosa.

### Bifidogenic effect of GOS in chicken

The largest abundance of *Bifidobacterium* spp. was found in caecum, and of *Lactobacillus* spp., in ileum. This finding is supported by Bjerrum et al. [55], who identified *Lactobacillus* as the predominant species in chicken ileum, whereas caecum was inhabited by more diverse microbial communities, including *Bifidobacterium* spp. The primary and continuous source of *Lactobacillus* spp. in ileum is the crop, which is almost exclusively colonised by this genus (relative abundance within 97–98%) throughout the chicken lifespan [56]. From the crop, *Lactobacillus* spp. is shed down the GIT and inhabits the small intestine, becoming the predominant species, especially in the ileum. Intestinal microflora starts developing in perinatal period, but its most rapid growth and diversification increases with age, during environmental exposition of the host [57]. Caecal microflora are more numerous and diverse compared to that from the upper guts and its function reflects the large intestine in mammalian species [57]. It is colonised by obligately anaerobic bacteria, such as *Clostridia*, which successively exclude *Lactobacillus* spp. [56].

*In ovo* delivery of GOS prebiotic into the chicken embryos increased the relative abundance of *Bifidobacterium* spp. in the caecum and decreased the relative abundance of *Lactobacillus* spp. in the ileum. Competitive exclusion of *Lactobacillus* spp. can be attributed to bifidogenic effect of GOS prebiotic. GOS used in this study was obtained from lactose by β-galactosidase activity of *Bifidobacterium bifidum* NCIMB 41171 [43]. For this reason GOS specifically promotes growth of *Bifidobacterium* spp. [43]. Due to the complex carbohydrate structure of GOS, it passes the upper GIT without degradation [58]. The genome of *Bifidobacterium* spp. encodes carbohydrate-degrading enzymes with high affinity to GOS [59]. Jung et al. (2008) found that in-feed supplementation with high proportion of GOS (12%) significantly increased faecal lactobacilli and bifidobacteria counts [36]. GOS supplementation and *Salmonella* spp. challenge diverged microbiome diversity of the caecal content, but those differences disappeared by day 40 post-infection [60]. GOS delivered in ovo on day 12 of egg incubation increased the lactobacilli and bifidobacteria faecal counts of 1-day-old chicks [28]. Here we shown the effects of microbiota modulation with *in ovo* stimulation in adult broiler chickens.

### Gene expression in jejunum and caecum

We have found that the administration of GOS had the most pronounced effects on gene expression regulation in jejunum and caecum. Both jejunum and caecum are of particular importance in avian GIT [61]. Most of the digestive and absorptive functions take place in jejunum. Jejunum is also the heaviest part of the small intestine. Even though nutrient absorption is a key physiological process, it happens quite rapidly—chyme passage time along jejunum is from 40 to 60 min.Due to availability of nutrients, jejunum is also target for both commensal bacteria and enteric pathogens. Shokker et al (2011) found correlation between *Salmonella* infection and jejunal gene expression [62]. Functions of jejunum in developing birds was characterized based on transcriptomic modulation; by day 21 post-hatching it was assigned to immune regulation [63]. These results are in line with this study in which we found immune-related gene expression that formed a jejunal cluster. Caecum is very distinct from jejunum in its function and physiology [9]. It takes part in electrolyte and water absorption but also allows for prolonged retention of chyme from two sources—jejunum and colon [9]. As such, it is caecum that is colonised by the majority of chicken intestinal microflora, which conveys numerous beneficial effects to the host [9]. Caecal gene expression been widely studied in chicken. Volf et al. (2017) compared germ-free, mono-associated and fully colonised chickens and determined that there was significant correlation between caecal microbiome and immunoglobulin expression [64]. Rychlik et al. (2014) reviewed the gene expression modulation in chicken caecal mucosa upon *Salmonella* challenge [65]. The authors confirmed the TLR pathway of bacterial recognition and pinpointed genes highly inducible in response to the enteric infection, such as metaloproteinases or cytokines. Based on transcriptomic caecal data, Higgins et al. (2011) inferred that probiotics clear *Salmonella* infection by inducing apoptosis in the caecal cells [66].

### Cytokine genes

*In ovo* stimulation with GOS increased mRNA expression of *IL-1β*, *IL-10* and *IL-12p40* cytokine genes in jejunum and caecum. In gut health, *IL-1β* plays dichotomous role, exerting both classic pro-inflammatory as well as protective functions [67]. The transient and low expression of *IL-1β* mediates beneficial effects in the intestinal mucosa and manifests itself in faster healing of epithelia in case of damage. Intestinal *IL1-β* is expressed constitutively in lamina propria cells and secreted into intestinal lumen in a condition of intestinal health as well as disease [68]. The latter drastically increases *IL-1β* abundance, making this cytokine a key mediator of intestinal inflammation [68]. *IL1-β* expression is not only modulated by pathogenic bacteria but also by healthy gut microbiome [69]. Animals primed with healthy microflora mount higher abundance of *IL-1β* in the guts in comparison to animals with sterile guts [69]. Commensal microbiota inhabiting the guts stimulate macrophages residing in lamina propria to express *pro-IL-1β*, which is an inactive form of *IL-1β* [70]. Activation of *pro-IL-1β* by cleavage into *IL-1β* is executed by inflammasome [71]. On the other hand, presence of inflammasome guarantees maintaining microbiome and gut homeostasis [71]. In this study mRNA abundance of *IL-1β* was enhanced by *in ovo* delivered GOS and this effect was related to the intestinal fragment. Hereby we claim that the treatment primed gut mucosa towards immunostimulation.

In the literature regarding intestinal homeostasis, the synthesis of pro-inflammatory cytokine *IL-12p40* is often compared to the level of anti-inflammatory cytokine *IL-10* [72]. It allows determination of the *IL-10/IL-12p40* ratio, which is indicative of the immunoregulatory (anti-inflammatory) vs. immunostimulatory (pro-inflammatory) function exerted by host-microbiome interaction in the guts [72]. Some probiotic strains are able to skew the *IL-12p40*/*IL-10* ratio towards either of the cytokines, which can either boost immune response and activate the pro-inflammatory cascade or limit it and thus prevent the damage that excessive inflammation can do to the host. This model reflects cell polarization by luminal agents like probiotics [73]. We have previously demonstrated that *in ovo* stimulation with different prebiotics and synbiotics led to down-regulation of the immune-related gene expression in caecal tonsils [31, 74, 75]. Haghighi et al. (2008) also found multi-strain probiotic given to chicks on day of hatching reduced *IL-12* gene expression in caecal tonsils of chickens challenged with *Salmonella* [76]. On the other hand, Kogut et al. (2013) demonstrated that feeding a peptide isolated from Brevibacillus texasporus probiotic to the chickens primed their immune responses to consecutive *Salmonella* challenge [77]. The dynamics between intestinal flora, environmental impact and immune status of the host is not completely understood. Maintaining the balance between activation and suppression of the immune responses upon microflora modulation will probably be a key point in the future research.

### Barrier function genes

HDP belong to broad-spectrum and evolutionary-conserved innate immunity effector molecules, referred to in the past as antimicrobial proteins [78]. HDP expressed by intestinal mucosa take part in intestinal innate immunity and mucosal defence [79]. Intestinal HDP are bound with mucins, creating a firm immunological and mechanical barrier between the host and the intestinal antigens [79]. Their major function is antimicrobial activity, but HDP also play other roles, including immunomodulation, chemotaxis, or wound repair [80]. HDP gene expression depends on microbial modulation. Abkari et al. (2008) demonstrated that gene expression of β-defensins and cathelicidins was triggered by *Salmonella* challenge in cecal tonsils broiler chicks[81]. The same genes were suppressed to the negative control level by earlier oral supplementation with multi-strain probiotics. The authors attribute such modulatory effects of probiotics on HDP gene expression to competitive exclusion of *Salmonella* by probiotic strains [81]. Other authors also found relation between intestinal HDP gene expression microbiota composition, including and *Salmonella* [82] or *E*. *coli* [64] challenge[64]. [82]

Mucins contribute to maintaining gut-barrier function and protect from enteric pathogens. Mucins are secreted into intestinal lumen and form a protective layer, which is resistant to proteolysis [83]. Goblet cells produce mucins in reaction to microbial stimuli. Prebiotics can alter the composition of the intestinal microflora, which in turn stimulates goblet cells towards mucin production [84]. In this study, GOS delivered *in ovo* mediated increase in *MUC6* expression in mucosa of jejunum and cecum. These results are in concordance with Bogucka et al. (2017), who demonstrated that GOS delivered *in ovo* increased the number of goblet cells in jejunum and ileum (caecum was not analysed) of 35-day-old broiler chickens [41]. Chicken mucin has confirmed *in vitro* cytotoxic activity against *Salmonella* [85]. Gut-barrier failure in chickens is associated with impaired expression of *MUC2*, a major intestinal gel-forming mucin [86]. Improved expression of *MUC2* under LPS challenge was mediated by *Bacillus*-based probiotics [87]. Smirnov et al. (2006) also found *in ovo* feeding with carbohydrates (applied intraamniotically on day 17.5 of egg incubation) increased Goblet cells number and *MUC2* expression in jejunum [88].

Integrity of intestinal mucosa is formed with epithelial cells connected with TJ proteins [89]. Intestinal permeability and its interaction with gut microbiota has been reviewed by Bischoff et al. [90]. Dysbiosis, defined as microbial imbalance, reduces intestinal integrity of the intestinal epithelia. When it happens and the host-microbiome barrier becomes faulty, intestinal antigens and lipopolysaccharides (LPS) leak into millieu of the body and cause inflammatory responses. Loosened TJ strands are major cause of the “leaky guts” [89]. Claudins constitute a key component of the TJ strand. They bind peripheral membrane proteins, including scaffold proteins, for example, *zonula occludens* protein 1 (ZO-1) [91]. Tight junction associated protein 1, encoded by the *TJAP1* gene, is incorporated later in time into the TJ strand, after the claudin-based junction has been established [92]. Enteric pathogens such as *Salmonella* or *Campylobacter* often target integrity of the tight junctions [93]. On the other hand, prebiotics and probiotics are known to stimulate expression of different TJ genes [87], including claudins [94]. *CLDN-1* encodes a barrier-forming claudin. Its increased expression leads to tightening the epithelial cells.

### Nutrient sensing genes

Intestinal nutrient sensing takes part in regulating gut hormones release based on gut content. Proteins encoded by *FFAR2* and *FFAR4* regulate glucose and lipids metabolism [95]. In particular, they mediate the release of anorectic gut hormones (incretin hormones) from epithelia into the intestinal lumen [96]. One of those incretins, glucagon‐like peptide‐1 (GLP‐1), regulates energy uptake via the brain-gut axis [96]. GLP-1 is produced by the intestine right after nutrient ingestion to promote satiety and decrease further feed uptake [96]. Down-regulation of *FFAR4* in duodenum of GOS suggests that GLP-1 activity was inhibited. Kolodziejski et al. [97] determined that GOS-based synbiotic delivered *in ovo* significantly reduced mRNA expression of *GLP-1* in duodenal content and GLP-1 protein in blood serum of adult broiler chickens. Down-regulation of GLP-1 pathways via *FFAR* could be one of the mechanisms explaining increased body weight in chickens stimulated *in ovo* with GOS [98].

*FFAR* genes serve also as SCFA receptors. *FFAR2* encodes GPR43, which is a receptor of SCFA, such as propionate, acetate and butyrate [99], end products of fibre fermentation by intestinal bacteria. *FFAR4* encodes GPR120, which recognizes long-chain unsaturated fatty acids, including omega-3, which are usually of dietary origin [100]. Increase in both, *FFAR2* and *FFAR4* along the GIT (except duodenum) suggests increased FFA content. SCFA are not only energy source but they can also regulate inflammatory immune responses in the guts [101]. Activation of *FFAR2* and *FFAR4* by free fatty acids leads to increased cytokine production by intestinal epithelial cells [95, 101]. It was also demonstrated that SCFA activates chicken monocyte/macrophage cell line (HD11) [102]. By connecting metabolic and immune effects, *FFAR* genes are important biomarkers of the intestinal microflora activity.

Glucose transporters take part in the absorption of glucose through the membrane of epithelial cells in a passive way, i.e., down the gradient of glucose concentration. *GLUT1* is a high-affinity constitutive glucose transporter. *GLUT2* is a low-affinity intestinal glucose co-transporter. It has been manifested that expression of *GLUT2* is activated by butyrate, which is one of the SCFA produced by intestinal microflora [103]. Also, studies on gnotobiotic mice showed that the presence of *Clostridium ramosum* activated *GLUT2* expression in jejunum [104]. However, the exact mechanisms are still unknown. Kolodziejski et al. [97] found that the digestive ability of duodenum was improved by *in ovo* delivery of GOS-based synbiotic, which manifested itself by an increase in trypsin and lipase activity in duodenal chyme, followed by dampening 70% of the activity of the amylase (*P* < 0.05). Based on this finding we hypothesize that there was less glucose available in duodenum as a source of carbon, which could have inhibited expression *GLUT2* and *GLUT5* in GOS-stimulated group.

## Conclusions

This paper provides insights into the mechanisms of the beneficial effects of the *in ovo* stimulation with GOS prebiotic in chickens. The effects were analysed on microbial and mucosal sites of the chicken GIT. Microbial stimulation with GOS delivered *in ovo* was manifested by increased abundance of *Bifidobacterium* spp. in caecum, and decreased abundance of *Lactobacillus* spp. in ileum. Mucosal gene expression was modulated mainly in jejunum and caecum. GOS delivered *in ovo* increased expression of the cytokine genes, barrier function genes and free fatty acid receptors. Varied expression of the glucose transporter genes indicated that GOS induced also modulation of energy metabolism. In conclusion, *in ovo* stimulation induced long-term, beneficial effects on the chicken gut homeostasis.

## Supporting information

S1 File qPCR dataset for calculating (A) bacterial and (B) gene abundances.
(XLSX)
